# Renoprotective Effect of Yiqi Yangyin Huayu Tongluo Formula against Diabetic Nephropathy in Diabetic Rats

**DOI:** 10.1155/2018/4276052

**Published:** 2018-12-06

**Authors:** Feng-li Wang, Yue-hua Wang, Lin Han, Hai-yan An, Jiang-hua Zhang, Xue-yun Zhang, Zhi-qiang Chen, Jian-guo Qin

**Affiliations:** ^1^Central Laboratory, Dongfang Hospital, Beijing University of Chinese Medicine, Beijing 100078, China; ^2^Department of Nephropathy, Hebei Medical University Third Hospital, Shijiazhuang, Hebei 050081, China; ^3^School of Basic Medicine, Beijing University of Chinese Medicine, Beijing 100029, China; ^4^Department of Nephropathy, Dongfang Hospital, Beijing University of Chinese Medicine, Beijing 100078, China; ^5^School of Integrated Traditional Chinese and Western Medicine, Hebei Medical University, Shijiazhuang, Hebei 050017, China; ^6^Department of Nephropathy, Hebei Provincial Hospital of Traditional Chinese Medicine, Shijiazhuang, Hebei 050017, China

## Abstract

Diabetic nephropathy is developed in 20-40% of patients with diabetes mellitus, and patients with diabetic nephropathy require dialysis and renal transplantation. Traditional Chinese medicine has been widely used in treating patients with diabetic nephropathy in China. However, the detailed mechanisms of traditional Chinese medicine remain unclear. Yiqi Yangyin Huayu Tongluo formula (ZY formula) is a traditional Chinese medicinal formula. Here, we demonstrated kidney protective effect of ZY formula on the rats with diabetic nephropathy. The therapeutic effect of ZY formula on the diabetic nephropathy was almost the same as that of Irbesartan, which proved to have excellent curative effects on diabetic nephropathy. We also demonstrated the mechanism of ZY formula effect on the diabetic nephropathy. First, we validated that the activation of ROS-JNK signaling pathway in diabetic rats could be reduced by ZY. Furthermore, collagen I expression could be downregulated by ZY formula treatment. Meanwhile, cell apoptosis in the kidney of diabetic rats could be alleviated by ZY formula.

## 1. Introduction

Diabetic nephropathy (DN), a leading cause of end-stage renal disease worldwide, is the main microvascular complication of type II diabetes mellitus [1]. DN is developed in 20-40% of patients with diabetes mellitus, and patients with DN require dialysis and renal transplantation [2]. Thus, clinical strategies for DN prevention will be in urgent need of improving [3]. The pathogenesis of DN is complicated. The characteristic of DN is the persistent albuminuria, accompanied by a progressive elevation of blood pressure, a reduction in glomerular filtration rate, and a reduced risk of cardiovascular events [4]. Both the slowdown of the progression toward end-stage renal disease (ESRD) and the decrease of glomerular filtration rate are closely related to the reduction of albuminuria level. Therefore, the end point for renoprotection could be indicated by the decline in albuminuria [5].

According to the theory of traditional Chinese medicine, it is commonly believed that the pathogenesis of DN is the lack of Yin and Qi. Many Chinese medicine formulas have been reported to be of great clinical efficacy in improving DN, because of the supplement of Yin and Qi from traditional Chinese medicine [6]. The extracellular matrix accumulation of mesangial cells [7, 8], signaling pathway in the renal cell [9, 10], and diabetes-related glomerular cell apoptosis [11] have widely been reported to be influenced by traditional Chinese medicine. Compared with other Chinese patent medicines available, Yiqi Yangyin Huayu Tongluo formula used in the present study is more innovative and reasonable treatment aimed at the pathogenesis of both Qi and Yin deficiency and blood stasis in DN.

A hallmark of DN is the extensive accumulation of extracellular matrix (ECM) in the interstitium and glomeruli [12], which leads to tubule-interstitial and glomerulosclerosis fibrosis and basement membrane thickening [13]. Collagen is an important component of ECM. Up to now, 28 types of collagen have been described and identified. The most common types of collagen are type I, type II, type III, type IV, and type V [14]. The expression of ECM proteins in renal cells is quite different. Type I collagen is expressed in mesangial cells and glomerular epithelial cells, but not glomerular endothelial cells and tubular epithelial cells [15]. During the progression of DN, the components of mesangial matrix, including fibronectin, type VI, V, and IV collagen, as well as other ECM components, such as type II and I collagen, that do not exist in glomeruli in the physiological situation, are extensively accumulated [16]. In particular, the staining study has proved that type IV collagen is increased in patients with DN [17] and the increased urinary excretion of type IV collagen is believed to reflect renal overproduction of this extracellular matrix proteins in early DN [18].

Enhanced oxidative stress is one of the main signaling pathways mediated during DN [19]. The balance between different antioxidant defense systems, including superoxide dismutase, dicarboxylic aldehyde, nicotinamide adenine dinucleotide phosphate, and glutathione peroxidase, determines the extent of oxidative stress [20, 21]. TGF-*β* expression is upregulated by ROS via MAPK signaling pathway and the nuclear receptor PPAR-*γ* [22, 23]. Many antioxidant treatments have been reported to be of great therapeutic effect on DN [24, 25].

Renal cell apoptosis, which leads to the functional regression of the kidney, is closely related to the progression of DN [26]. Caspase-3 is the major mediator of cell apoptosis and the activation of caspase-3 is mediated by proteolytic cleavage of its prodomain [27]. The mitochondrial apoptosis pathway in renal cells is always activated by hyperglycemia and the induced levels of cleaved caspase-3 can be examined in the kidney of rats with DN [28].

Here, we found that the therapeutic effect of ZY formula to reduce injury in the STZ-induced DN model is almost the same as that of Irbesartan. The renal injury caused by DN can be alleviated by ZY formula through downregulating the level of ROS, ECM accumulation, and apoptosis in the kidney

## 2. Materials and Methods

### 2.1. Experimental Animals

A total of 40 healthy male SD rats (body weight 120-150g, 4-5 weeks) were supplied by Laboratory Animal Center of Hebei province (Certificate No. 1008095). All experimental procedures were approved by the Ethnic Committee of Hebei Medical University.

### 2.2. Pharmacological Agents

ZY formula is composed of eight different Chinese herbs, namely,* Astragalus membranaceus* (2g),* Rehmannia *(2g),* Salvia miltiorrhiza *(1.8g),* Ligusticum wallichii *(1.3g),* Rhizoma polygonati* (3g),* Lumbricus *(1g),* Leech *(1.5g),* Buthus martensi kirsch *(1g), which were all gifted by Yifang Company (Guangdong, China). The mixed mass ratio for the above eight Chinese herbs was 4:4:3.6:2.6:3:1:1.5:1 and then water was added to adjust the final concentration to 0.3g/ml. The dosage of each rat was given according to the human equivalent dose (HED), d_B_ = d_A_ × R_B_ × W_A_^1/3^ ÷ (R_A_ × W_B_^1/3^), where d_B_ is dosage for rat, d_A_ is dosage for adults human (1g/Kg), R_B_ is coefficient of rat body type (0.09), W_A_ is body weight of adult human (60 Kg), R_A_ is coefficient of human body type (0.11), and W_B_ is body weight of rat. The quality of ZY formula was controlled according to the Chinese medicine prescription granule fingerprint map. Our previous study has shown that ZY formula has a good clinical effect on early DN [29]. The positive agent, Irbesartan, was purchased from Sanofi Aventis Pharma Ltd (Hangzhou, China). Irbesartan was dissolved in saline solution.

### 2.3. Establishment of DN Model and Treatment

Before our experiments, totally 40 SD rats were fed at least one week for adaption. Next, the rats in the normal group were fed with a regular diet and other 30 rats received a high-fat diet (Experimental Animal Center of Hebei Medical University, Hebei, China) for 12 weeks. The high-fat diet contains 18.6% protein, 24.3% fat, 5.4% ash, 2.5% fiber, 6.5% moisture, and 45.0% carbohydrate, and the caloric content of food was 4.36kcal/g. Then these 30 rats were intraperitoneally injected of streptozocin (STZ) at 30 mg/kg, but the rats of normal control group were injected with 0.1M natrium citricum buffer solution (pH=4.5) of the same volume. 72 h after the injection, serum glucose level was detected in all rats. When the serum glucose level was 16.7 mmol/L, the diabetic rat model was successfully established. Then, diabetic rats were divided into the model group, Irbesartan group, and ZY group randomly, with 10 rats in each group. Rats injected with saline solution were considered as the model group (n=10). Rats treated with Irbesartan solution (11.51mg/kg/day) were considered as the Irbesartan group. Rats of ZY group were treated with ZY formula (1g/kg/day). Rats in different groups were observed for 32 weeks. 32 weeks later, all rats were sacrificed.

### 2.4. Histopathological Examination

10% neutral formalin phosphate buffer was used to fix the renal tissues. Next, the renal tissues were dehydrated through alcohol, embedded into the paraffin, cut into 2 *μ*m sections, and stained with hematoxylin-eosin (HE), periodic acid-Schiff (PAS), and Masson. All the sections were examined by light microscopy. Renal tissues of rats were cut into 1 mm cubes and then fixed in 4% glutaraldehyde for an hour. Next, the samples were fixed in 1% osmium tetroxide and dehydrated by graded alcohol series. The samples were embedded in Epon, cut with ultramicrotome (Leica, German), and stained with lead citrate and uranyl acetate. The sections were examined with a Hitachi H-7500 transmission electron microscope. The degree of glomerulosclerosis was calculated by glomerulosclerosis index (GSI), defining that glomeruli (at a magnification of ×400) in each kidney was graded in accordance with their severity of glomerular damage using a similar scoring system (0-4 grades) as described previously [30]. And the renal interstitial fibrosis index (RIFI) was calculated semiquantitatively according to the ratio of positive area to the area of glomeruli (0, negative; 1, positive < 10%; 2, positive 10% ~ 25%; 3, positive 25% ~ 50%; and 4, positive > 50%).

### 2.5. Quantitative Real-Time Polymerase Chain Reaction Analysis

Primers used for detection of mRNA were as follows: collagen I: forward 5′- TCCTGCCGATGTCGCTATC-3′ and reverse 5′-CCATGTAGGCTACGCTGTTCTTG-3′; GAPDH: forward 5′-GTTACCAGGGCTGCCTTCTC-3′ and reverse 5′-GGGTTTCCCGTTGATGACC-3′; caspase-3: forward 5′-GGAGCTTGGAACGCGAAGAA-3′ and reverse 5′-ACACAAGCCCATTTCAGGGT-3′. Renal cortical tissues were collected carefully and were frozen at −80°C for extraction of total RNA by Trizol reagent (Beibo Biotechnology Company, Shanghai, China). Next, cDNA was synthesized from total RNA using the SuperScript™ III First-Strand Synthesis SuperMix for qRT-PCR (Invitrogen, USA) according to the instructions of the manufacturer. Expression of genes was analyzed by quantitative real-time PCR (qPCR). qPCR reaction mixture contained 10 *μ*L qPCR mix, 1 *μ*L 10 *μ*M forward and reverse primers, and 2 *μ*L cDNA, and the volume was brought to 20 *μ*L with RNase-free water. The reaction condition was optimized so as to pre-denature at 95°C for 2 min, denature at 94°C for 20 s, anneal at 60°C for 20 s, and extend at 72°C for 30 s for 40 cycles. The RNA quantity was normalized against the GAPDH content, and gene expression was quantified according to the 2^−△Ct^ method.

### 2.6. Flow Cytometry Analysis of ROS

For exogenous ROS study, single cells of renal tissues were suspended in ice-cold PBS buffer. ROS was measured by staining the cells (Beyotime Biotechnology Company, Jiangsu, China) according to the manufacturer's protocol. After staining, cells were strained briefly and analyzed using Epics-XL II (Beckman-Coulter, USA). The intensity of ROS production by each individual cell was represented by mean fluorescence intensity (MFI).

### 2.7. Western Blot Analysis

Renal cortical tissues were collected carefully and were frozen at −80°C. Proteins for Western blot were extracted by lysing with RIPA buffer from the renal cortex. After centrifugation, the supernatant was collected. Proteins (35*μ*g) were resolved in 12% sodium dodecyl sulfate polyacrylamide gel (SDS-PAGE) and transferred to the PVDF membrane. The anti-JNK antibody, anti-p-JNK antibody, anti-*β*-actin antibody, and anti-caspase-3 antibody were all purchased from Santa Cruz (California, America). *β*-actin was used as the loading control.

### 2.8. Statistical Analysis

All the data in this study were shown as mean ± SD and were analyzed by using one-way ANOVA analysis in the SPSS 13.0 (SPSS Inc., USA). The differences between the two groups were analyzed by using SNK assay. The differences were considered statistically significant only when the value of *P* was less than 0.05.

## 3. Results

### 3.1. The Level of Urinary Protein is Reduced by ZY Formula Treatment

**Figure 1:**
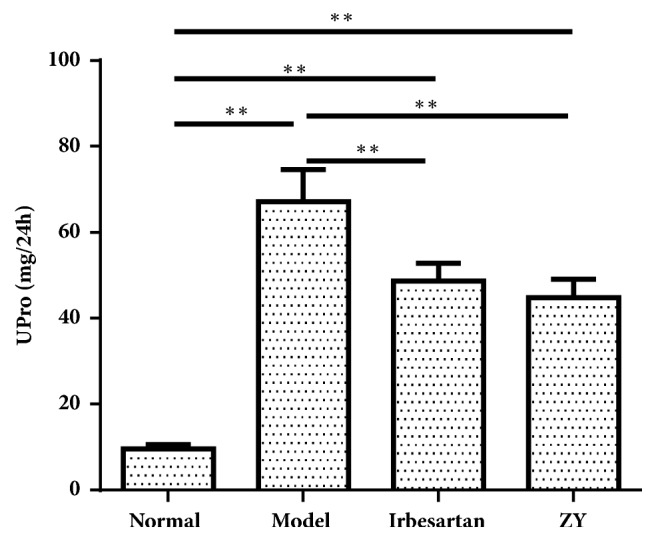
**Level of urinary protein is reduced by ZY treatment**. Changes in the level of urinary protein after treatment. Data were expressed as mean values (±SD) from 10 rats. Normal, SD rats; Model, STZ (30mg/kg) treated group; Irbesartan, STZ + Irbesartan (11.51mg/kg) treated group; ZY, STZ + ZY (1.0g/kg) treated group. *∗*p<0.05, *∗∗*p<0.01.

To validate whether the DN of rats could be induced by STZ and the effect of ZY formula on the regulation of urinary protein, we first examined the level of urinary protein of all the rats. The level of urinary protein of rats with DN induced by STZ was much higher than those of normal rats over the 13-week experimental period (Figure 1). The elevated levels of urinary protein were significantly reduced by ZY formula and Irbesartan treatment from 32 weeks onwards at the doses of 1 g and 11.51mg/ kg BW/ day. The progression of DN could be slowed down by ZY formula and Irbesartan. Irbesartan had been reported to be of great therapeutic effect on DN. Those rats treated with Irbesartan were set to the positive control. The level of urinary protein between the ZY group and Irbesartan group did not show significant difference (Figure 1). This meant the effect of ZY formula and Irbesartan in reducing urinary protein level of diabetic rats was similar.

### 3.2. Renal Ultrastructural Changes in Rats with DN Can Be Reversed by ZY Treatment

**Figure 2:**
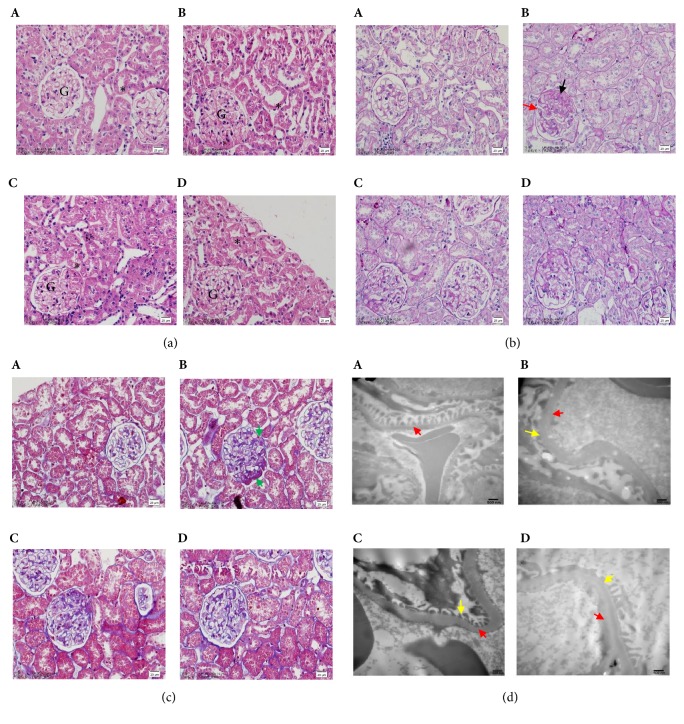
**Renal ultrastructural changes in rats with DN can be reversed by ZY treatment**. Renal pathological changes in rats with DN after treatment. (a–c) Effects of ZY formula on renal pathology changes in diabetic rats, as assessed by HE staining (400× magnification), PAS staining (400× magnification), and Masson staining (400× magnification). (d) Morphology change in the kidney (transmission electron microscopy. Magnification: 20,000×). Representative microphotographs of the kidney are shown for the normal group (A); model group (B); Irbesartan group (C); and the ZY group (D). G: glomerulus. *∗*:  renal tubule. Black arrow: mesangial matrix. Red arrow: glomerular basement membranes (GBM). Green arrow: fibrosis. Yellow arrow: foot process.

Next, we examined the histological changes in the kidney of rats. Light microscope was used to examine the histology of kidney in the rats with DN. In HE stains, there were no obvious abnormalities in glomerular and tubular structures of the kidneys in rats of normal group. However, there were significant mesangial expansion, tubular dilatation, and slightly glomerular hypertrophy in rats of DN model group. However, both after 32 weeks of ZY formula and Irbesartan treatment, the changes in both glomerular and tubular structures induced by STZ were significantly reversed, compared with that of saline treated rats (Figure 2(a)). PAS staining clearly revealed thickening of glomerular basement membranes (GBM) and increase of mesangial matrix as compared with controls, which were reversed by ZY formula and Irbesartan treatment (Figure 2(b) and Table 1). Masson trichrome showed increased intensity of fibrosis, indicating accumulation of extracellular matrix proteins (Figure 2(c) and Table 1). Next, the morphometric differences between different groups were analyzed by electron microscope. Compared with the normal rats, significant thickness of GBM, fusion of foot process accompanied with lager width of foot process, was observed in the rats with DN. After the rats with DN were treated with ZY formula and Irbesartan, the changes in the fusion of foot process can be reversed significantly but without significant change in GBM (Figure 2(d)). These results suggest that the histological changes induced by DN can be reversed by ZY formula and Irbesartan.

### 3.3. The ROS-JNK Signaling Pathway in the Kidney

**Figure 3:**
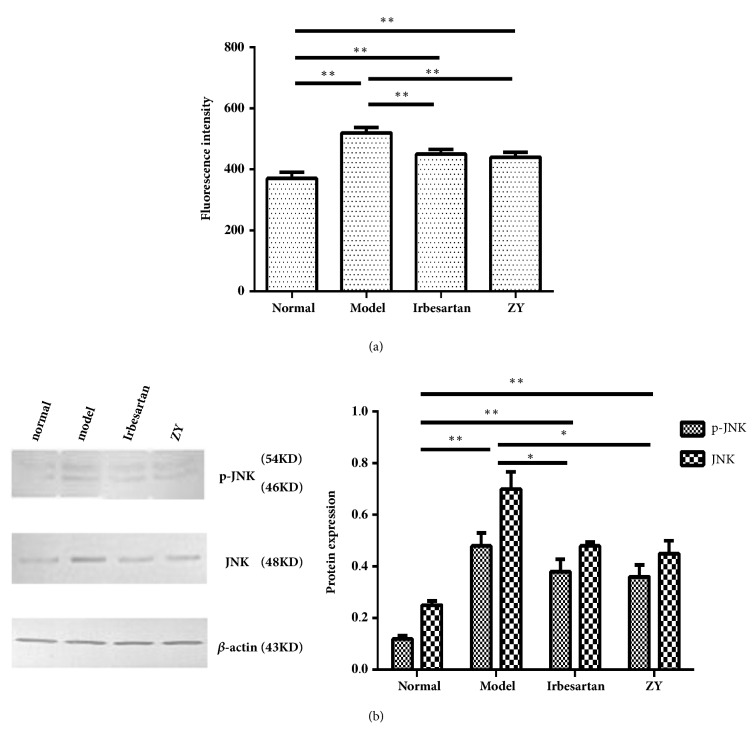
**The ROS-JNK signaling pathway in the kidney**. Effects of ZY formula and Irbesartan on ROS-JNK signaling pathway in DN model rats. (a) Effects of ZY formula and Irbesartan on ROS level in the kidney. (b) Effects of ZY formula and Irbesartan on the expression of JNK and phosphorylated JNK in the kidney. The Western blot results were presented on the left and the semiquantitative results determined by WB were presented on the right. SD rats were divided into normal group, model group, Irbesartan group, and ZY group. *∗*p<0.05, *∗∗*p<0.01.

ROS production in the kidney was always induced by high blood glucose. Exposure to the STZ, the ROS production in the kidney of rats, was significantly upregulated. However, ZY formula and Irbesartan partially reduced STZ-induced ROS production. No significant difference was seen on the level of ROS production between ZY and Irbesartan treatment (Figure 3(a)).

MAPK signaling pathway could be activated by ROS. JNKs belonged to the MAPK family and played an important role in the MAPK pathway. The JNK gene expression correlated greatly with the development of type II diabetes [31, 32]. According to the Western blot analysis, the levels of both JNK and phosphorylated JNK proteins in the kidney of rats with DN were higher than in the kidney of normal rats. Treatment of DN with either ZY formula or Irbesartan significantly reduced the diabetic activation of JNK (the level of phosphorylated JNK) (Figure 3(b)). These results suggested that ROS and JNK pathway could be induced in the rats with DN, but ZY formula could suppress the diabetic activation of both ROS and JNK pathway.

### 3.4. Effect of ZY Formula on Collagen I Expression

**Figure 4:**
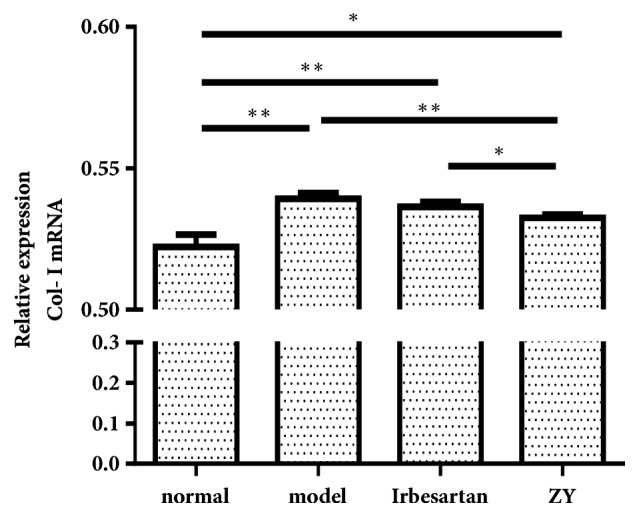
**Effect of ZY treatment on collagen I expression**. Effects of ZY formula and Irbesartan on the expression of collagen I in DN model rats. The mRNA level of collagen I in the kidney was examined by real-time PCR. SD rats were divided into normal group, model group, Irbesartan group, and ZY group. *∗*p<0.05, *∗∗*p<0.01.

We next wanted to examine whether ZY formula had an effect on the accumulation of extracellular matrix (ECM). The results showed that the mRNA level of collagen I was higher in the diabetic model group, compared with the normal group. After treatment with ZY formula, the expression of collagen I obviously decreased, compared with the diabetic model group and Irbesartan group (Figure 4). This result suggested that activation of collagen I expression in the rats with DN could be reversed by ZY formula treatment.

### 3.5. ZY Protected Kidney of Diabetic Rats from Apoptosis

**Figure 5:**
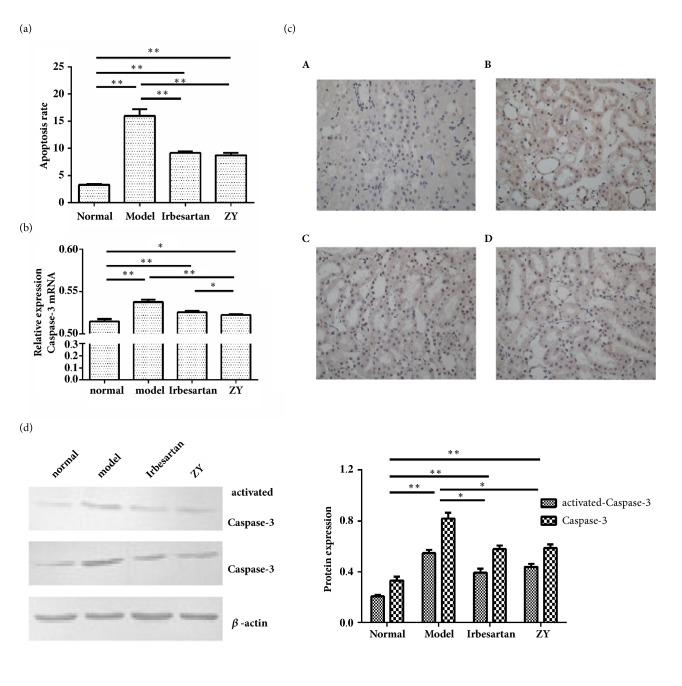
**ZY protected kidney of diabetic rats from apoptosis**. ZY formula protected kidney of diabetic rats from apoptosis. (a) The level of renal cells apoptosis was measured by flow cytometry assay and the data were expressed as mean ±SD. *∗*p<0.05, *∗∗*p<0.01. (b) The mRNA level of caspase-3 was examined by real-time PCR. The data were expressed as mean ±SD. *∗*p<0.05, *∗∗*p<0.01. (c) The expression of caspase-3 in the kidney was examined by immunohistochemically staining (400× magnification). (d) Western blot was used to examine the expression of caspase-3 and cleaved caspase-3. The Western blot results were presented on the left and the semiquantitative results determined by WB were presented on the right. *∗*p<0.05, *∗∗*p<0.01. SD rats were divided into normal group, model group, Irbesartan group, and ZY group.

To determine the antiapoptotic effect of ZY formula, flow cytometry assay was performed. The apoptosis rate of renal cortex cells was significantly increased compared with normal rats. The treatment of both ZY formula and Irbesartan was effective in reducing apoptosis in diabetic rats. There was no significant difference between the ZY group and Irbesartan group (Figure 5(a)).

The activation of caspase-3 was closely related to the activation of apoptosis pathway. In diabetic rats induced by STZ, the quantitative real-time PCR examination (Figure 5(b)) and immunohistochemically staining (Figure 5(c) and Table 2) for caspase-3 revealed that strong PCR signaling and immunoreactivity of caspase-3 were detected in the kidney. The caspase-3 expression in the kidney was restored in diabetic rats treated with ZY formula. Next, the cleaved caspase-3 was also detected by Western blot (Figure 5(d)). These results showed that the active forms of caspase-3 were increased in the kidney of rats with DN, but could be restored after treatment with ZY formula and Irbesartan. These results suggested that renal cell apoptosis induced by STZ could be reduced by ZY formula treatment.

## 4. Discussion

DN is a kind of progressive disease that causes injury of kidney, with progression over time [33]. In the early stage of DN, there are some pathological changes in the kidney that can be examined, such as mesangial matrix accumulation, enhanced oxidative stress, and renal cell apoptosis. In our study, we found that ZY formula had a great effect in reducing the ECM accumulation, ROS, and cell apoptosis in the kidney, which means that early syndrome of DN could be alleviated and the progression of DN could be inhibited by ZY formula.

The TGF-*β* signaling pathway is believed to contribute to a predominant role in the progression of DN. Most of the traditional Chinese medicine or medicinal formulas have a great effect on the TGF-*β* signaling pathway. Tangshen formula protected the kidney of diabetic rats from diabetic injuries by reducing the expression of TGF-*β* receptor I and TGF-*β* [34]. The renal injury in diabetic rats could be reduced significantly by She Jing Xiao Bai capsule and the mechanism involved was the decrease of CTGF and TGF-*β* expression induced by She Jing Xiao Bai capsule treatment [35]. In addition, the lipid metabolism and TGF-*β* signaling pathway could be affected by Bebeerine [36] and Gui Qi mixture [37], which led to the recovery of kidney in DN rats. However, in our study, we found that the JNK signaling pathway was affected by ZY formula. After the rats were induced by the STZ, the JNK signaling pathway in the kidney was activated. When the diabetic rats were treated with the ZY formula, the activation of JNK signaling pathway could be reduced in the kidney. Most of the traditional Chinese medicinal formulas consisted of different herbs in order to improve the therapeutic efficacy and reduce side effects. Thus, many signaling pathways can be affected by these traditional Chinese medicinal formulas. Due to the important role of TGF-*β* signaling pathway and the multiple targets of traditional Chinese medicine, the influence of ZY formula on TGF-*β* signaling pathway or other signaling pathways needs to be further examined. Also, in our future work, the major active components of ZY formula responsible for the renoprotection should be identified.

The accumulation of extracellular matrix (ECM) in glomeruli is one of the most remarkable hallmarks of DN [38]. Type IV collagen is the major component of the basement membranes of kidney [39]. Some of the traditional Chinese medicines, such as Tangshen formula [34] and Danggui-Shaoyao-San [33], protected kidney from renal injury through regulating the accumulation of extracellular matrix in glomeruli. Although the upregulation of type IV collagen expression is the main cause of ECM in glomeruli [40], the roles of type I collagen during the progression of DN should not be neglected. Type I collagen does not exist in glomeruli in the physiological situation. But during the progression of DN, type I collagen starts to accumulate in glomeruli which results in the renal fibrosis and renal function loss [16]. Type I collagen seems to be a biomarker of DN progression. In our study, we found that type I collagen expression could be affected by ZY formula treatment. But the accumulation of ECM in glomeruli is caused not only by the production of ECM associated protein, but also by the inhibition of ECM associated protein degradation. How ZY formula affects the ECM metabolism needs further examinations.

Reactive oxygen species (ROS) release in the kidney was one of the characteristics of DN progression [41]. The ROS generation and oxidative modified proteins production were increased in mitochondria of renal tubule cells of rats after the induction of STZ [42]. In the kidney of rats with DN, the SOD activity was decreased and the MDA level was upregulated [43]. ROS generation in the kidney could be inhibited by Curcuma aromatic [44]. The SOD activity was increased and MDA level was reduced after the treatment of Fufang Xue Shuan Tong capsules [45], Crocin [46], and Luteolin [47] in diabetic rats and then inhibited the progression of DN. We found that the ROS generation in diabetic rats could be inhibited by ZY formula. The process of ROS generation in the kidney is complicated, so the mechanism of ZY formula to affect ROS production needs to be further examined.

## 5. Conclusion

The therapeutic effect of ZY formula to reduce renal injury in the STZ-induced DN model was almost the same as that of Irbesartan. The renal injury caused by DN could be alleviated by ZY formula through downregulating the ROS generation, ECM accumulation, and renal cells apoptosis in the kidney.

## Figures and Tables

**Table 1:** Quantification of PAS (GSI) and Masson (RIFI) staining.

Group	GSI	RIFI
Normal group	0.12 ± 0.01	0.13 ± 0.01
Model group	1.20 ± 0.04^a^	0.53 ± 0.03^a^
Irbesartan group	0.87 ± 0.04^ab^	0.37 ± 0.02^ab^
ZY group	0.81 ± 0.03^ab^	0.35 ± 0.03^ab^

^a^p<0.05 vs. normal group

^b^p<0.05 vs. model group

^c^p<0.05 vs. Irbesartan group

**Table 2:** Quantification of caspase-3 staining.

Group	Percentage area (%)
Normal group	4.53 ± 0.44
Model group	13.72 ± 0.38^a^
Irbesartan group	7.91 ± 0.35^ab^
ZY group	8.11 ± 0.41^ab^

^a^p<0.05 vs. normal group

^b^p<0.05 vs. model group

^c^p<0.05 vs. Irbesartan group

## Data Availability

No data were used to support this study.
